# Effectiveness of Video-Feedback with Cognitive Preparation in Improving Social Performance and Anxiety through Super Skills for Life Programme Implemented in a School Setting

**DOI:** 10.3390/ijerph17082805

**Published:** 2020-04-18

**Authors:** Mireia Orgilés, Silvia Melero, Iván Fernández-Martínez, José Pedro Espada, Alexandra Morales

**Affiliations:** Department of Health Psychology, Miguel Hernández University, 03202 Elche, Spain; smelero@umh.es (S.M.); i.fernandez@umh.es (I.F.-M.); jpespada@umh.es (J.P.E.); alexandra.moraless@goumh.umh.es (A.M.)

**Keywords:** anxiety symptoms, children, video-feedback, Super Skills for Life, social performance, school

## Abstract

Effectiveness of video-feedback with cognitive preparation to treat anxiety problems (especially social anxiety) has been scarcely explored on children. Super Skills for Life (SSL) is a CBT-based intervention to reduce anxiety and comorbid problems that, apart from social skills training and behavioural activation, integrates video-feedback with cognitive preparation. This study aimed to evaluate SSL effects, implemented in a school setting, on social performance and to test self-concept and social skills as potential mediators of pre- and post-test changes in social anxiety and generalized anxiety. Sample comprised 57 children aged 8–11 years with emotional symptoms. Children were video recorded in the first and last session to assess social performance. Anxiety and self-concept measures were completed by children pre-test and post-test. Participants reduced anxiety behaviours and improved social and communication skills after treatment. In general, girls showed better social performance than boys, but SSL impact was greater in males. Social self-concept was the only mediator of change in pre- to post-treatment social anxiety. This study provides evidence of SSL to improve children’s social performance and reduce anxiety through video-feedback with cognitive preparation. Improving social concept seems essential to reduce social anxiety. An SSL programme is an ideal prevention protocol for anxious children.

## 1. Introduction

Anxiety disorders are among the most common psychological problems in children and adolescents. Recent studies claim that approximately 9–46.7% of minors suffer from anxiety symptoms, which implies a great assistance demand in the mental health centres [1,2,3]. If childhood-onset anxiety disorders are untreated, they tend to become chronic [4] and the risk of developing other anxiety disorders, depression, and substance abuse in adulthood increase considerably [5,6]. In addition, these disorders cause impairment in the academic, social, and family settings and entail a high cost in health, educational, and social care services [1,7,8].

Among anxiety disorders in childhood and adolescence, generalised anxiety and social anxiety are the most prevalent and persistent [3]. Generalised anxiety is characterized by a feeling of permanent stress and worry, which can impair social functioning [9]. Fear of negative evaluation in social situations (social anxiety) is highly common in populations with generalised anxiety. Children who suffer social anxiety show a deficit of social skills [10] and have a negative perception of their social performance [11]. Other studies suggest that, when examining social interaction behaviours separately, socially anxious children do not differ from others in their social skills (e.g., gaze, smile, voice, etc), but by showing more nervous behaviours (e.g., trembling, stumbling over words, stuttering, self-manipulations, etc) [12]. When anxious children enter a social situation, negative rumination activates a public self-image which threatens their social self-concept [12,13]. Spence, Donovan, and Brechman-Toussaint [14] found that socially anxious children tended to anticipate more negative outcomes and reported poorer expected performance on social tasks. Social anxiety reduces the quality of social interactions and children’s social, academic, and emotional self-concept [15]. Given that anxious children show impairments in peer relations [16], early detection and treatment are key to improve their social performance and prevent social rejection.

Regarding gender differences, the literature indicates that anxiety disorders are more prevalent in girls [2,3]. Most of the research on social anxiety have found that girls report higher levels of socially anxious symptoms than boys [3,17,18]. Furthermore, girls’ friendships are more impaired by social anxiety than are boys’ [17,19]. Despite this, studies indicate that males show greater signs of nervousness in their social performance, and females have better social skills and non-verbal behaviour (e.g., eye contact, nodding, and smiling), even if they have social anxiety [20,21]. The effectiveness of social anxiety interventions has also been examined in terms of gender, finding a higher improvement in girls’ anxious symptoms, social skills, and other associated problems [18,20].

Cognitive-behavioural therapy (CBT) is the first-choice treatment for childhood anxiety [22,23]. Most studies of CBT-based programmes have been conducted in research settings, such as university clinics, with more highly qualified and experienced professionals than the average clinicians [24]. Thus, concern about generalising results to real-world settings leads researchers to suggest applying interventions in school contexts, which are more familiar and natural to children [25,26]. This would also reduce the stigma of receiving treatment in mental health clinics [25]. Combining effective cognitive-behavioural techniques, behavioural activation, social skills training, and video-feedback with cognitive preparation, Essau and Ollendick [27] developed Super Skills for Life (SSL), a transdiagnostic prevention protocol for children with anxiety and comorbid problems (e.g., depression, low self-esteem, and lack of social skills) that can be implemented in different contexts.

Generalised and social anxiety symptoms are particularly addressed in SSL, because video-feedback with cognitive preparation is an effective CBT-based technique to contrast children’s negative thoughts [11,28,29,30,31], increasing their confidence and reducing their anxiety in the following speech tasks [32]. In the SSL programme, children must perform in front of peers, facing a video camera, and assess their social performance. Cognitive preparation before viewing videos encourages children to create a mental image of their performance and to watch it objectively, which allows changing and improving their social performance appraisal [33]. The SSL programme includes video-taped role-play activities with which children learn social interaction skills with peers and problem solving, which they should practise as homework. Furthermore, to increase positive social experiences and enhance their social self-perception, children’s participation in rewarding activities is encouraged through different tasks based on behavioural activation.

SSL has been recently translated and culturally adapted for Spanish children and adolescents [34,35,36]. The Spanish version of SSL is effective in decreasing anxiety, children’s life anxiety interference, depression, negative self-esteem, and behavioural problems (e.g., peer and conduct problems). Despite the promising results of SSL, the evaluation of its effectiveness to improve social performance and communication skills through video-feedback with cognitive preparation is pending. Effectiveness of video-feedback with cognitive preparation to treat anxiety problems (especially social anxiety) has been widely explored in adolescents and adults, but few studies have focused on the child population. According to Essau et al. [20], more evidence is needed about the mechanisms underlying the effectiveness of video-feedback in SSL to reduce anxiety symptoms, including self-concept and social performance.

This study aimed to evaluate the effectiveness of video-feedback with cognitive preparation of the SSL programme, implemented in the school setting, through three objectives: (1) to analyse SSL effects through video-feedback with cognitive preparation on children’s social performance, and separately by gender; (2) to compare SSL effects on social performance behaviours (gaze, vocal quality, length, discomfort, conversation flow, micro-behaviours, nervous behaviours and global impression) between boys and girls; and (3) to study the dimensions of self-concept (social, academic, emotional, family, and physical self-concept) and social performance as mediators of change in social anxiety and generalised anxiety. Based on previous studies, it is hypothesised that: (1) children’s social performance will improve after treatment; (2) SSL’s effect on social performance will differ by gender; girls will present higher social performance than boys after treatment; and (3) social performance and self-concept areas (specifically, the social area) will mediate the change in pre- to post-treatment of social anxiety and generalised anxiety.

## 2. Materials and Methods 

### 2.1. Participants

**Figure 1:**
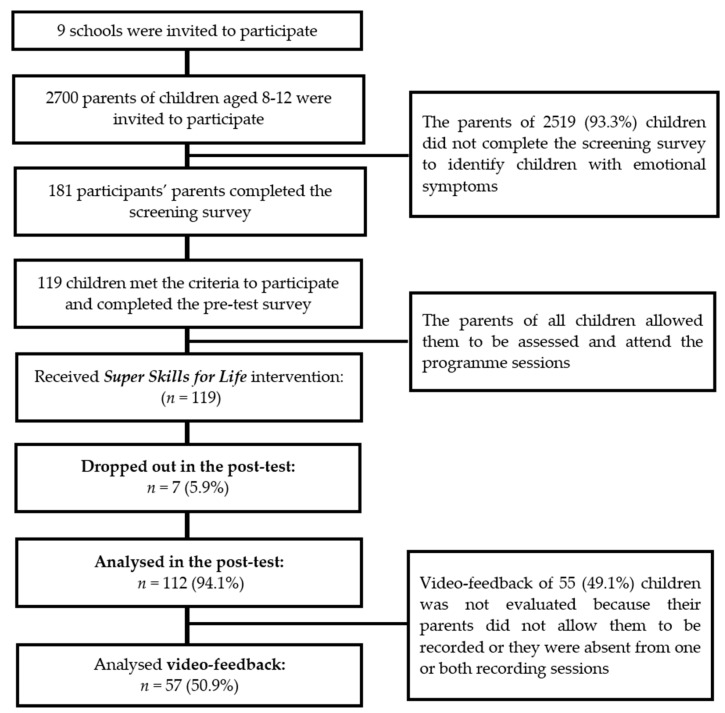
Progress of children participating in the trial.

In the original study, sample was made up of 112 children. Of these participants, a subsample of 50.89% (*n* = 57) children were assessed for improvements in social performance using video-feedback with cognitive preparation (Figure 1). The final sample in the present study comprised 57 children (68.4% were males) aged between 8 and 11 years (*M* = 9.35, *SD* = 1.15). The age distribution was as follows: 8 (*n* = 20; 35.1%), 9 (*n* = 8; 14%), 10 (*n* = 18; 31.6%), and 11 (*n* = 11; 19.3%). Participants were recruited from 9 schools located in the southeast of Spain. Practically all the participants were Spanish-born (98.2%), except for one (1.8%) who was born in the United States, but all of them were Spanish-speaking. The mean number of siblings was 1.16 (*SD* = 1.17).

Given that parents tend to report more global information about their children’s emotional state [37], they completed the Strengths and Difficulties Questionnaire-Parent version (SDQ-P) [38] to screen their children’s emotional symptoms. Children who showed emotional symptoms based on scores equal to or greater than 4 on the Emotional symptoms subscale of the SDQ-P were selected to participate in the study. Another inclusion criterion was that the child had not received previous psychological or pharmacological treatment for emotional problems. Sample was evaluated at baseline (Session 1) and immediately after receiving the intervention (Session 8).

### 2.2. Measures

Sociodemographic variables were obtained by a set of items that evaluated age, gender, school year, number of siblings, and birthplace of participants.

The AF-5: Self-Concept Form 5 [39] measures five dimensions of self-concept: Social (performance in social relationships); Academic/Professional (role as a student/worker); Emotional (perception of emotional state in general and in specific situations); Family (participation and integration in the family unit); and Physical self-concept (appearance and physical state). It consists of 30 statements that are rated from 1 to 99 according to the degree of agreement with the content of each statement. Cronbach’s alpha was 0.85 in the current study.

The Screen for Child Anxiety Related Emotional Disorders [40] assesses anxiety disorder symptoms in children and adolescents. This instrument consists of 41 items grouped into five subscales: Somatic/Panic; Generalised Anxiety; Separation Anxiety; Social Anxiety; and School Phobia. Respondents rate on a 3-point scale (0 = almost never; 1 = sometimes; 2 = often) the frequency with which they experience each symptom. The total score is obtained by summing the relevant items. In this study, the Social Anxiety and Generalised Anxiety subscales were used for mediation analyses. The Cronbach’s alpha for this sample was 0.89 for the total score, 0.66 for Social Anxiety, and 0.73 for Generalised Anxiety.

The Social Performance Rating Scale [41] evaluates behavioural indicators of anxiety in a videotaped social performance. The scale consists of five dimensions: Gaze; Voice Quality; Length; Discomfort; and Conversation Flow. The total score is obtained by combining the 5 dimensions. In the current study, items were adapted to the 2-min speech task (there was no conversation partner). Observers were trained according to the guidelines provided by Fydrich et al. [41]. The internal consistency was adequate (α = 0.70) for this sample.

The Objective Performance Questionnaire [12] requires an observer to rate the child’s performance during the 2-min speech task. This scale contains eight items grouped into three dimensions: Micro-Behaviours (displayed during the social situation), Nervous Behaviours (how comfortably the child performs in front of the camera) and Global Impression (overall opinion of the performance). Each item is scored on a four-point Likert scale ranging from 1 (not very much) to 4 (very much). The scale had adequate reliability (α = 0.75) in this study.

The Strengths and Difficulties Questionnaire—Parent version (SDQ-P) [38] was only used for study participant selection purposes. It is a brief screening questionnaire for assessing children’s and adolescents’ psychological adjustment. The 25 items in the SDQ comprise 5 scales of 5 items each: Emotional Symptoms; Conduct Problems; Hyperactivity/Inattention; Peer Problems; and Prosocial Behaviour. Items are rated on a 3-point scale ranging from 0 (Not true) to 2 (Certainly true). In this study, only the Emotional Symptoms subscale score was analysed for participant selection. Following the original 3-band categorization, a cut-off score ≥4 was chosen as a selection criterion, which includes the categories borderline and abnormal. Cronbach’s alpha of the SDQ-P was 0.76 in this study.

### 2.3. Procedure

This study was approved by the Institutional Review Board (IRB) at the Miguel Hernández University in Spain (DPS.MO.02.14). Nine primary schools in southeast Spain were randomly selected, and their headmasters were invited to participate in the study. The headmasters agreed to collaborate by sending a letter to the students’ families with information about the study. Interested parents completed an online form that served as a screening tool. Meetings with parents of the selected children were held to provide information about the programme, confidentiality, and voluntary participation, and also to complete the informed consent. 

As noted, SSL was delivered at the children’s schools in the afternoons. Facilitators were therapists specialized in child and adolescent psychology and with at least 2 years’ experience. All of them received intensive training in the SSL programme before its implementation and were given a leader’s manual with a detailed description of each session. Weekly meetings with facilitators were held to discuss possible problems during the implementation and to collect data. Besides, each facilitator recorded in writing the degree of application of each session, which allowed assessing the implementation fidelity.

The videos’ rating through the social performance scales (see measures section) was carried out by two doctoral students trained as observers. Each observer independently rated the 2-min speech tasks of each participant at pre- and post-intervention. The discrepancies between them were discussed until an agreement on the scores was reached. Raters were blind to other children’s scores in other measures and did not take part in the programme implementation. The final rating scores were used to evaluate the effectiveness of the intervention to change social performance outcomes in the speech task.

### 2.4. Super Skills for Life (SSL) Programme

The SSL is a transdiagnostic CBT-based programme aimed to treat emotional problems and their comorbid symptoms [27]. Through the programme, children learn to identify and manage their own and others’ emotions, cognitive restructuring skills, behavioural activation, relaxation techniques, social and communication skills, and problem-solving strategies. The SSL intervention consisted of eight weekly 45-min sessions in small groups comprising 6–8 children. Sessions 1 and 2 involve teaching children skills to enhance their social performance and making a 2-min speech facing a video camera. In the first session, they had to imitate a television presenter giving information about themselves (name, favourite food, animal, and hobby). In the eighth session, as an exercise of the generalization of the skills learned, they had to talk like a TV presenter in front of the camera about which SSL activities they liked the most and which skills they considered most useful for their life. Before watching their recordings, the children were instructed to attend to their social behaviours and not focus on how they felt during the speech. Subsequently, the children watched their recordings and were requested to assess their real social performance. Orgilés et al. [34] provide a detailed description of the Spanish version of SSL.

### 2.5. Statistical Analyses

The sample was described using frequencies (percentages) and means (standard deviation) of the sociodemographic variables. Gender differences in main outcomes and sociodemographic variables were studied and controlled in the analyses. Cohen’s *d* was calculated to report effect size for statistically significant differences. Because the measurement scales were ordinal, Spearman’s rank order coefficient was used to test inter-rater reliability (IRC). These analyses provided evidence of the extent to which individual evaluators’ rating pointed in the same direction. An intent-to-treat perspective was applied; therefore, the number of sessions attended by participants was not a criterion to be included in the analyses. Attrition analyses were included to identify possible differences between children who were selected for the current study and the rest (*n* = 55). As recommended by Rosenthal and Rosnow [42], pre-specified trial hypotheses were planned and were tested as follows: (1) a contrast compared post-test with pre-test in the entire sample, (2) a contrast compared post-test with baseline scores only for girls, (3) a contrast compared post-test with baseline scores only for boys, and (4) a contrast compared post-intervention scores by gender to detect whether SSL shows effects differentially for boys and girls. Short-term effects of SSL to improve social performance outcomes were evaluated using generalised estimating equations (GEE), adjusting for gender, age, baseline differences, and clustering in participating schools [43,44]. All analyses were controlled for participating school, age, gender (boys were coded as 1 and girls were coded as 2), and baseline scores. Estimated marginal means, adjusted odds ratio (AOR) and their 95% confidence intervals (CI) are reported. All analyses were performed using SPSS v.25 (IBM Corp., Armonk, NY, USA). Following Essau et al. [20], we studied whether the incremental scores of social performance and self-concept (including academic, social, emotional, familial, and physical areas) were potential mediators of change between pre- and post-treatment social anxiety and generalised anxiety symptoms. Mediation analyses using SPSS PROCESS v3.0 macro [45] were conducted. We calculated 95% bias-corrected bootstrap confidence intervals (5000 samples) for indirect effects. The significant criterion was *p* ≤ 0.05.

## 3. Results

### 3.1. Attrition 

Regarding attrition analysis, differences between selected children for this study and those who were not selected were only found in gender (*AOR* = 0.40, *CI* [0.19, 0.85], *p* = 0.01). A higher percentage of males completed the post-intervention survey compared to females (68.4% vs. 31.6%); however, most of the participants were males. No differences were found in age (*p* > 0.05), number of siblings (*p* > 0.05), anxiety (SCARED) (*p* > 0.05), generalised anxiety (*p* > 0.05), social anxiety (SCARED subscale) (*p* > 0.05), or social self-concept (AF-5 subscale) (*p* > 0.05) between the two groups (selected and non-selected children for the study). The mean number of sessions that children attended was 7.52 (*SD* = 0.78: range: 1–8). For the current sample, there were no gender differences in the sociodemographic variables.

### 3.2. Inter-Judge Reliability

Table 1 shows the correlation coefficients of the two independent observers’ ratings for all the SPRS (Social Performance Rating Scale) and OPQ-C (The Objective Performance Questionnaire) subscales. The concordance index for these calculations was above 0.90 pre-test and 0.95 post-test, which indicates excellent inter-rater reliability on all subscales. These results show high agreement between the raters in the recording assessments.

### 3.3. Behavioural Changes in the Speech Task

Children’s social performance was evaluated through the 2-min speech task pre- and post-treatment. As shown in Table 2, all the participants improved their scores after the intervention in all the SPRS subscales, and in the total score; as well as in all the OPQ-C subscales. As shown in Table 3, this within-subject improvement in the different subscales of social performance was statistically significant, with *p*-values ranging from 0.005 to <0.001, except for the subscale Length (*p > 0.05*). Therefore, children reduced their anxious behaviours and increased their overall social and communication skills from pre- to post-treatment.

### 3.4. Gender Differences

Before the intervention, a significant between-subject effect of gender was obtained. At baseline, girls presented higher scores in Conversation Flow (*p* < 0.05, *d* = 0.73) and Nervous Behaviours compared to boys (*p* < 0.05, *d* = 0.63). Furthermore, Discomfort was marginally significant (*p* > 0.05), which meant that girls felt more comfortable than boys during their social performance.

In girls, significant effects were obtained after treatment in four of the five SPRS dimensions (except for Conversation Flow), in the total SPRS score, and in all three OPQ-C variables (Table 3). Boys also improved significantly in four of the five SPRS dimensions (except for Length), in the total SPRS score and all three OPQ-R variables (*p* < 0.05 to *p* < 0.001) (Table 3). 

Gender differences in social performance were observed post-test. Boys reduced their Nervous behaviours more than girls (*p* < 0.01) and showed higher scores in Discomfort (*p* < 0.05) (Table 3). Given that higher scores on the Discomfort scale indicate greater comfort, these results show that, after treatment, boys felt more comfortable during their social performance than did girls.

### 3.5. Mediators of Change

Table 4 shows the 95% confidence intervals for the mediating effects. Social self-concept was the only significant mediator of change from the pre- to post-test Social Anxiety subscale (M = −0.36, 95% CI [−0.65, −0.08]). Mediating effects were not found for the rest of the variables. None of the potential mediators was significant for change from the pre- to post-test Generalised Anxiety subscale.

## 4. Discussion

The main objective of the study was to examine the effects of the SSL video-feedback with cognitive preparation on children’s social performance and its relationship with social anxiety and generalised anxiety in a Spanish sample of schoolchildren with subclinical anxiety symptoms. The SSL has proven to be an adequate programme for decreasing anxiety symptoms and improving mood, self-esteem, and social performance in primary school children aged 8–12 in the United Kingdom [20] and in Spain [34]. 

In this study, the use of video-feedback with cognitive preparation has provided benefits in children’s social performance, enhancing their prosocial behaviours and communication skills, and reducing signs of discomfort in these social situations. These findings are consistent with previous studies, in which children and adolescents presented fewer anxiety symptoms, improved their performance in the speech task, and increased their self-confidence [11,26,32]. Moreover, video-feedback is a useful component to modify children’s negative appraisal of their social performance, because children who suffer from social anxiety show a tendency to self-rate themselves as socially unskilled [12,14].

The ratings of the 2-min speech task by observers at pre- and post-treatment indicated that the children’s social performance improved significantly after the SSL intervention at school. In the current study, children showed fewer behavioural indicators of anxiety (such as inadequate movements, stiffness, self-manipulation, stuttering, stumbling over words etc.), and greater self-assurance when speaking, their voice volume and tone were more appropriate, their speech fluency and coherence had improved, and they showed adequate gaze and friendliness in their second speech. In the study of Essau et al. [20], similar results were found at 6-month follow-up on behavioural indicators of anxiety. However, our results suggest that the programme effects are noticeable from the end of the intervention, as in other studies conducted in the school setting [26].

Although social performance improved in the entire sample in the post-intervention evaluation, SSL impact was greater in males. Compared to girls, boys showed significant improvements in behavioural measures associated with displaying discomfort and nervousness. These results contradict previous literature, as it has been found that interventions in social anxiety showed a greater impact on girls [18]. Despite this, according to the study of Essau et al. [20], girls obtained better ratings in almost all the variables of social performance, specifically in gaze, length, conversation flow, micro-behaviours, and global impression. This may be because girls had better social performance at baseline and also due to a ceiling effect. Although girls improved their performance after the programme, it was difficult to find significant differences between the two time points. Therefore, our working hypothesis is partially fulfilled because, after treatment, girls showed better social performance, although they also continued to manifest greater anxious behaviours than boys. These findings agree with previous literature, which states that girls are socially more skilled, but also suffer more social anxiety than boys, and this is associated with social functioning problems [2,17,19,26].

Social self-concept mediated the effect of SSL on social anxiety symptoms. According to previous studies, social self-concept of individuals with social anxiety is negatively affected by a highly distorted public self-image [14,15,28]. Video-feedback with cognitive preparation, as a component of the SSL, contributed to children’s disconfirming their negative beliefs about their social performance and adjusting their self-image during the speech [29,31]. As suggested in the literature, [11,29,33], during the programme, participants were instructed to view the videotape neutrally, as if they were watching a real television presenter. Their own evaluation as external observers in the first speech increased their self-confidence and made their predictions for the second speech more positive [32]. Therefore, it is not surprising that the improvement of social self-concept through the SSL programme has a beneficial impact on reducing social anxiety symptomatology. In line with the study of Essau et al. [20], social performance was not a mediator for pre- to post-test change in the Social Anxiety and Generalised Anxiety subscales. Thus, the third hypothesis of this study was partially confirmed. Results agree with the findings of Cartwright-Hatton et al. [12], who argued that children maintain social anxiety through their negative beliefs about their social performance, regardless of their social skills. Future studies should explore other potential mediators of SSL effectiveness, for example, the children’s cognitive emotion-regulation strategies or the presence of other comorbid problems, such as depression or behavioural problems.

### Limitations

The results of the current study need to be interpreted considering several limitations. First, sample size was relatively small, which makes it difficult to generalize the results. Future studies including a larger sample should be carried out to confirm the results and analyse differences by age and gender. Second, there was no control group with which to compare the obtained findings. Randomized controlled trials are needed in future research to incorporate untreated children’s outcomes to provide more evidence of the programme’s effect [26]. Third, there are no data on the self-assessment of the children’s social performance, but only on the objective rater’s evaluation. An inclusion of the children’s perspective in future research would be interesting to analyse changes in the appraisal of their own social performance [20,33].

## 5. Conclusions

In summary, despite the above limitations, this study provides evidence of the positive effects of video-feedback with cognitive preparation in the SSL programme to improve children’s social performance and reduce anxious symptomatology. In addition to clinical improvements, at an educational level, enhancing social performance can contribute to reducing peer rejection and increase the quality of social interactions [16]. Improvement of social self-concept seems a key to reduce social anxiety. Thus, these findings suggest that interventions aimed at treating social anxiety should include components that enhance children’s social self-concept. The SSL methodology makes the programme an ideal prevention protocol for children with anxiety problems, especially those with social anxiety, which can be applied in both clinical and school contexts. These results support the use of transdiagnostic approaches, which include different strategies based on CBT for the treatment of anxiety and social performance problems in children and contribute to understanding the transportability of a clinical intervention into real-world school settings. Therefore, this research provides strategies to promote children’s mental health that are useful for the development of public health policies.

## Figures and Tables

**Table 1:** Spearman correlations for inter-judge reliability.

Outcomes	Pre-Test	Post-Test
**SPRS ^1^**		
Gaze	0.99	0.99
Vocal quality	0.96	0.95
Length	0.98	0.97
Discomfort	0.95	0.95
Conversation flow	0.90	1
Total	0.95	0.99
**OPQ-C ^2^**		
**Micro-behaviours**	0.98	0.97
1. How loud and clear was the child’s voice?	0.99	0.96
2. How much did the child look at the camera?	0.97	0.97
3. How much did the child smile	0.97	0.98
**Nervous**	0.98	0.99
4. How nervous did the child look?	0.98	0.97
5. Did the child stumble over the child’s words?	1	1
**Global**	0.97	0.98
6. How clever did the child look?	0.93	0.98
7. How friendly did the child look?	0.99	0.97
8. How good was the child’s speech?	0.95	0.98

^1^ Social Performance Rating Scale; ^2^ The Objective Performance Questionnaire.

**Table 2:** Estimated marginal means (95% confident interval) of the outcomes pre-test and post-test by gender.

Outcomes	Sample	Pre-Treatment	Post-Treatment
**SPRS ^1^**			
Gaze	Girls	3.48 (3.39, 3.57)	4.04 (3.69, 4.39)
	Boys	3.54 (3.47, 3.61)	3.87 (3.61, 4.13)
	Total	3.51 (3.45, 3.57)	3.96 (3.74, 4.17)
Vocal quality	Girls	3.67 (3.53, 3.80)	4.05 (3.77, 4.34)
	Boys	3.61 (3.51, 3.71)	4.25 (4.06, 4.44)
	Total	3.64 (3.55, 3.72)	4.15 (3.98, 4.32)
Length	Girls	3.78 (3.69, 3.87)	4.11 (3.88, 4.34)
	Boys	3.71 (3.64, 3.78)	3.81 (3.55, 4.08)
	Total	3.74 (3.69, 3.80)	3.96 (3.79, 4.14)
Discomfort	Girls	3.50 (3.37, 3.63)	4 (3.70, 4.31)
	Boys	3.32 (3.24, 3.41)	4.30 (4.03, 4.56)
	Total	3.41 (3.33, 3.49)	4.15 (3.96, 4.34)
Conversation flow	Girls	3.83 (3.73, 3.94)	4 (3.68, 4.33)
	Boys	3.66 (3.58, 3.74)	3.99 (3.75, 4.23)
	Total	3.75 (3.68, 3.81)	4 (3.80, 4.20)
Total	Girls	18.21 (17.90, 18.51)	20.15 (19.20, 21.10)
	Boys	17.90 (17.67, 18.13)	20.28 (19.42, 21.14)
	Total	18.05 (17.87, 18.24)	20.22 (19.60, 20.83)
**OPQ-C ^2^**			
**Micro-behaviours**	Girls	8.18 (7.96, 8.39)	9.84 (9.14, 10.55)
	Boys	8.22 (8.11, 8.33)	9.32 (8.93, 9.72)
	Total	8.21 (8.10, 8.31)	9.49 (9.14, 9.38)
1. How loud and clear was the child’s voice?	Girls	2.88 (2.78, 2.98)	3.61 (3.33, 3.88)
	Boys	2.87 (2.79, 2.95)	3.59 (3.43, 3.74)
	Total	2.88 (2.81, 2.94)	3.60 (3.44, 3.75)
2. How much did the child look at the camera?	Girls	2.68 (2.59, 2.77)	3.12 (2.81, 3.43)
	Boys	2.71 (2.65, 2.76)	2.94 (2.70, 3.17)
	Total	2.69 (2.64, 2.74)	3.03 (2.83, 3.22)
3. How much did the child smile?	Girls	2.61 (2.50, 2.71)	3.11 (2.74, 3.48)
	Boys	2.64 (2.56, 2.71)	2.79 (2.58, 3)
	Total	2.62 (2.56, 2.68)	2.95 (2.74, 3.16)
**Nervous behaviours**	Girls	2.95 (2.83, 3.08)	2.62 (2.38, 2.86)
	Boys	3.01 (2.91, 3.11)	2.55 (2.34, 2.76)
	Total	2.98 (2.90, 3.06)	2.59 (2.43, 2.75)
4. How nervous did the child look?	Girls	2.02 (1.92, 2.13)	1.63 (1.39, 1.88)
	Boys	2.16 (2.10, 2.23)	1.29 (1.13, 1.46)
	Total	2.12 (2.06, 2.18)	1.40 (1.26, 1.54)
5. Did the child stumble over the child’s words?	Girls	1.18 (1.11, 1.26)	1.02 (0.92, 1.11)
	Boys	1.22 (1.15, 1.28)	1.01 (0.94, 1.08)
	Total	1.21 (1.16, 1.25)	1.01 (0.95, 1.07)
**Global impression**	Girls	8.62 (8.42, 8.81)	10.06 (9.58,10.55)
	Boys	8.45 (8.28, 8.62)	9.58 (9.04, 10.12)
	Total	8.54 (8.41, 8.66)	9.82 (9.46, 10.18)
6. How clever did the child look?	Girls	2.94 (2.85, 3.03)	3.33 (3.11, 3.54)
	Boys	2.84 (2.77, 2.91)	3.23 (3.02, 3.43)
	Total	2.89 (2.83, 2.95)	3.28 (3.13, 3.43)
7. How friendly did the child look?	Girls	2.88 (2.77, 2.99)	3.20 (2.99, 3.42)
	Boys	2.87 (2.78, 2.96)	3.43 (3.22, 3.65)
	Total	2.87 (2.80, 2.95)	3.32 (3.17, 3.47)
8. How good was the child’s speech?	Girls	2.80 (2.73, 2.87)	3.30 (3.07, 3.53)
	Boys	2.73 (2.66, 2.80)	3.14 (2.93, 3.35)
	Total	2.76 (2.72, 2.81)	3.22 (3.06, 3.37)

^1^ Social Performance Rating Scale; ^2^ The Objective Performance Questionnaire; Higher scores denote better social performance except for “Nervous behaviours” where higher scores indicate greater anxiety.

**Table 3:** Generalised linear models and effect size estimates for the intervention effect on the speech-task outcomes in the post-test (compared to the baseline) by gender.

Outcomes	Sample	Post-Treatment	
		AOR ^1^ (95% CI ^2^)	*p* Value
**SPRS ^3^**			
Gaze	Girls	1.74 (1.18, 2.55)	0.005
	Boys	1.39 (1.04, 1.86)	0.02
	Total	1.49 (1.18, 1.88)	0.001
Vocal quality	Girls	1.47 (1.01, 2.16)	0.04
	Boys	1.89 (1.47, 2.44)	<0.001
	Total	1.75 (1.41, 2.16)	<0.001
Length	Girls	1.39 (1.06, 1.82)	0.01
	Boys	1.10 (8.82, 1.48)	0.49
	Total	1.19 (0.95, 1.48)	0.11
Discomfort	Girls	1.64 (1.15, 2.34)	0.005
	Boys	2.64 (1.98, 3.56)	<0.001
	Total	2.28 (1.79, 2.89)	<0.001
Conversation flow	Girls	1.18 (0.80, 1.73)	0.39
	Boys	1.39 (1.06, 1.82)	0.01
	Total	1.32 (1.06, 1.65)	0.01
Total	Girls	6.99 (2.53, 19.29)	<0.001
	Boys	10.85 (4.30, 27.39)	<0.001
	Total	9.44 (4.63, 19.24)	<0.001
**OPQ-C ^4^**			
Micro-behaviours	Girls	5.29 (2.37, 11.78)	<0.001
	Boys	3.01 (1.96, 4.60)	<0.001
	Total	3.59 (2.43, 5.32)	<0.001
1. How loud and clear was the child’s voice?	Girls	2.05 (1.46, 2.88)	<0.001
	Boys	2.05 (1.67, 2.50)	<0.001
	Total	2.05 (1.72, 2.44)	<0.001
2. How much did the child look at the camera?	Girls	1.56 (1.09, 2.21)	0.01
	Boys	1.26 (0.98, 1.61)	0.07
	Total	1.34 (1.09, 1.65)	0.005
3. How much did the child smile?	Girls	1.64 (1.08, 2.49)	0.01
	Boys	1.16 (0.91, 1.48)	0.21
	Total	1.30 (1.04, 1.61)	0.01
Nervous behaviours	Girls	0.71 (0.52, 0.97)	0.03
	Boys	0.63 (0.47, 0.82)	0.001
	Total	0.65 (0.53, 0.81)	<0.001
4. How nervous did the child look?	Girls	0.67 (0.51, 0.89)	0.005
	Boys	0.41 (0.34, 0.51)	<0.001
	Total	0.48 (0.40, 0.57)	<0.001
5. Did the child stumble over the child’s words?	Girls	1.18 (1, 1.40)	0.05
	Boys	0.81 (0.71, 0.92)	0.002
	Total	0.82 (0.74, 0.91)	<0.001
Global impression	Girls	4.23 (2.80, 6.41)	<0.001
	Boys	3.09 (1.62, 5.86)	0.001
	Total	3.41 (2.15, 5.40)	<0.001
6. How clever did the child look?	Girls	1.47 (1.17, 1.84)	0.001
	Boys	1.46 (1.14, 1.89)	0.003
	Total	1.47 (1.22, 1.77)	<0.001
7. How friendly did the child look?	Girls	1.74 (1.38, 2.19)	<0.001
	Boys	1.39 (1.05, 1.84)	0.01
	Total	1.49 (1.21, 1.83)	<0.001
8. How good was the child’s speech?	Girls	1.64 (1.30, 2.07)	<0.001
	Boys	1.50 (1.18, 1.92)	0.001
	Total	1.55 (1.29, 1.86)	<0.001

^1^ Adjusted Odds Ratio; ^2^ Confidence Interval; ^3^ Social Performance Rating Scale; ^4^ The Objective Performance Questionnaire. Higher scores denote better social performance, except for “Nervous behaviours”, where higher scores indicate greater anxiety. Each analysis was adjusted for the baseline measure, gender, age, and school level.

**Table 4:** Results of confidence intervals for mediating effects.

	*M* ^1^	*SE* ^2^	Lower Limit	Higher Limit
**Social performance (OPQ-C)**				
(change in social anxiety scores)	0.01	0.04	−0.04	0.12
(change in generalised anxiety scores)	−0.0007	0.01	−0.03	0.02
**Academic self-concept**				
(change in social anxiety scores)	−0.09	0.08	−0.29	0.03
(change in generalised anxiety scores)	0.002	0.01	−0.04	0.02
**Social self-concept**				
(change in social anxiety scores)	−0.36	0.15	−0.65	−0.08
(change in generalised anxiety scores)	0.02	0.02	−0.01	0.07
**Emotional self-concept**				
(change in social anxiety scores)	0.004	0.02	−0.01	0.01
(change in generalised anxiety scores)	0.003	0.01	−0.02	0.03
**Familiar self-concept**				
(change in social anxiety scores)	−0.05	0.08	−0.23	0.07
(change in generalised anxiety scores)	0.002	0.01	−0.02	0.03
**Physical self-concept**				
(change in social anxiety scores)	−0.10	0.11	−0.34	0.10
(change in generalised anxiety scores)	−0.006	0.02	−0.04	0.04

^1^ Mean; ^2^ Standard Error
